# The Effects of Social Processing and Role Type on Attention Networks: Insights from Team Ball Athletes

**DOI:** 10.3390/brainsci13030476

**Published:** 2023-03-11

**Authors:** Noemi Passarello, Michela Mellone, Pierpaolo Sorrentino, Andrea Chirico, Fabio Lucidi, Laura Mandolesi, Francesca Federico

**Affiliations:** 1 Department of Humanities, University of Naples Federico II, 80133 Naples, Italy; noemi.passarello@unina.it; 2 Department of Social and Developmental Psychology, Faculty of Medicine and Psychology, University of Roma “Sapienza”, 00185 Rome, Italy; michela.mellone@uniroma1.it (M.M.); andrea.chirico@uniroma1.it (A.C.); fabio.lucidi@uniroma1.it (F.L.); francesca.federico@uniroma1.it (F.F.); 3 Institut de Neuroscience des Systemès, Aix-Marseille University, 13288 Marseille, France; ppsorrentino@gmail.com

**Keywords:** open-skill sports, social attention, attention network test, executive control, social stimuli

## Abstract

(1) Background: Several findings have shown how social stimuli can influence attentional processes. Social attention is crucial in team ball sports, in which players have to react to dynamically changing, unpredictable, and externally paced environments. Our study aimed at demonstrating the influence of social processing on team ball sports athletes’ attentional abilities. (2) Methods: A total of 103 male players divided by sport (soccer, handball, and basketball) and by role (striker, midfielder, or defender) were tested through a modified version of the Attention Network Test (ANT) in which they were exposed to both social and non-social stimuli. (3) Results: Social stimuli positively impacted the athletes’ abilities to focus on target stimuli and ignore conflicting environmental requests (t = −2.600, *p* = 0.011 *). We also found that the athletes’ roles impacted their performance accuracy. Specifically, differences were found in the ability to maintain a general state of reactivity between athletes (strikers vs. midfielders: t = 3.303, *p* = 0.004 **; striker vs. defenders: t = −2.820, *p* = 0.017 *; midfielders vs. defenders: t = −5.876, *p* < 001 ***). (4) Conclusion: These findings revealed that social stimuli are crucial for performance enhancement in team ball sports athletes. Further, we suggest that it is possible to draw specific attentional profiles for athletes in different roles.

## 1. Introduction

Attention can be defined as a multi-component ability that allows us to direct, sustain, and switch mental focus [1,2]. Selective information processing appears to be critical for cognitive functioning, as it enables us to respond appropriately to environmental changes and implement the most contextually appropriate behavior [3]. Different functional networks have been distinguished between attention components, including alertness to maintain a general state of reactivity in response to sensory stimulation (the alerting network), orientation to a subset of sensory information to be processed for privileged purposes (the orienting network), and executive control acting on post-sensory representations when there is competition for access to a limited-capacity central system (the executive-control network) [4,5].

Attentional processes are widely investigated in sports science, as they can strongly influence athletes’ performance, especially in sports where the context is constantly changing and athletes have to adapt their actions to frequent variability [6]. Several findings have shown marked associations between attentional abilities and levels of sports experience [7,8,9], as well as role type [10]. Fontani and collaborators [11] found differences in the attentional styles of high- and low-experienced athletes in reactivity sports (karate and volleyball). Specifically, they found that high-experienced athletes exhibited enhanced sustained attention and better working-memory abilities. Further evidence has shown that expert athletes used better attention allocation, a key aspect of behavioral control that allows the optimization of performance when confronted with conflicting stimuli by generating, maintaining, and adjusting sets of goal-directed processing tactics [6,12]. For example, athletes’ ability to focus their gaze on relevant information, while ignoring irrelevant information, has been associated with higher levels of performance [13]. Studies have proven that elite athletes reach their peak level of performance through gaze fixation on target stimuli before executing direct actions [14]. An extended final ocular fixation before movement initiation, the so-called “quiet eye”, allows athletes to achieve better performance, aiding optimal attentional control [13]. This process seems to be crucial for movement accuracy [15], a decisive competence in sports performance. Such an ability enables athletes to achieve optimal attentional control, even in stressful and competitive environments [7,8,13]. In this context, visual attention seems to be crucial for selecting the spatial elements that are relevant to correct behavior execution. Through saccadic eye movements, athletes can identify and focus on objects in the environment by rapidly moving the fovea towards the direction of stimuli [16]. The same process enables them to fix their attention on other players in the field, both teammates and opponents. By fixing their focus on the other player, an athlete can see where he/she is looking and anticipate his/her intention. The process by which we are able to infer another person’s attentional focus by the direction of their gaze is defined as social attention [17]. In a recent review, Federico [18] stated that social-information processing influences attentional abilities [19,20,21] and is an important factor in individuals’ adaptation to their surrounding environment. Social signals are primarily communicated through faces, and gaze-direction processing is crucial for socialization. As a result, social attention plays a very important role in regulating social relationships from very early on in life [22]. There is a great deal of reliance on this ability in team ball sports, in which players have to react to a dynamically changing, unpredictable, and externally paced environment [23,24,25].

Although all this evidence suggests that the social nature of stimuli has a major influence on athletes’ attentional processes, there is still no scientific evidence of this association. Our study aimed to fill this gap. To achieve this, we tested a sample of team ball sports (soccer, handball, and basketball) athletes playing in three different roles: one mainly based on defense (defenders), one mainly based on tactics (midfielders), and one mainly based on attack (strikers). A modified version of the Attention Network Test (ANT) [4,25] was administered to the entire sample with social and non-social stimuli. Findings from our study could serve to advance scientific knowledge about social attention engagement in sports practice and its use as a training tool in team ball sports.

## 2. Materials and Methods

### 2.1. Participants

We recruited a total of 103 male soccer, handball, and basketball amateur players (mean age = 19.91 ± 3.10) from Campania and Lazio sport associations. All athletes had at least 10 years of experience in their own sports (mean years of experience = 10.92 ± 2.39). We divided our sample into three experimental groups according to the type of role played by the athletes: the Defenders group (N = 32) was composed of defense-focused athletes; the Midfielders group (N = 32) was composed of tactics-focused athletes; and the Strikers group (N = 38) was composed by attack-focused athletes (Table 1). A priori power analysis conducted on GPower revealed that our sample was more than sufficient to obtain a partial eta squared = 0.04 with 80% power (F_(2,81)_ = 3.19; sample size suggested = 84).

The inclusion criteria were normal or corrected-to-normal vision and right-handedness. The exclusion criteria comprised current or past presence of psychopathology; psychiatric, neurological, or motor disorders; or other medical illness. The participants were voluntarily enrolled after written informed consent was obtained. The study was approved by the Local Ethics Committee of the “Federico II” University of Naples (protocol number: 11/2020) and was carried out in accordance with the Declaration of Helsinki.

### 2.2. Attention Network Test (ANT)

The Attention Network Test (ANT) [4] was designed according to the attention network approach. Under different conditions, this test allows the evaluation of alerting, orienting, and executive-control network functioning. In this study, we use a modified version of the ANT with social and non-social stimuli [25]. All features of the ANT protocol are described in the following subsections.

#### 2.2.1. Apparatus

Stimuli were presented on a 12-inch color monitor. A PC (OS = Windows 64 bit; RAM = 8.00 GB; CPU = 11th Gen Intel(R) Core(TM) i7-1165G7 @ 2.80GHz) running E-Prime software (version 2.0) controlled the presentation of stimuli, timing operations, and data collection. Responses were gathered with a standard computer mouse.

#### 2.2.2. Stimuli

**Figure 1:**
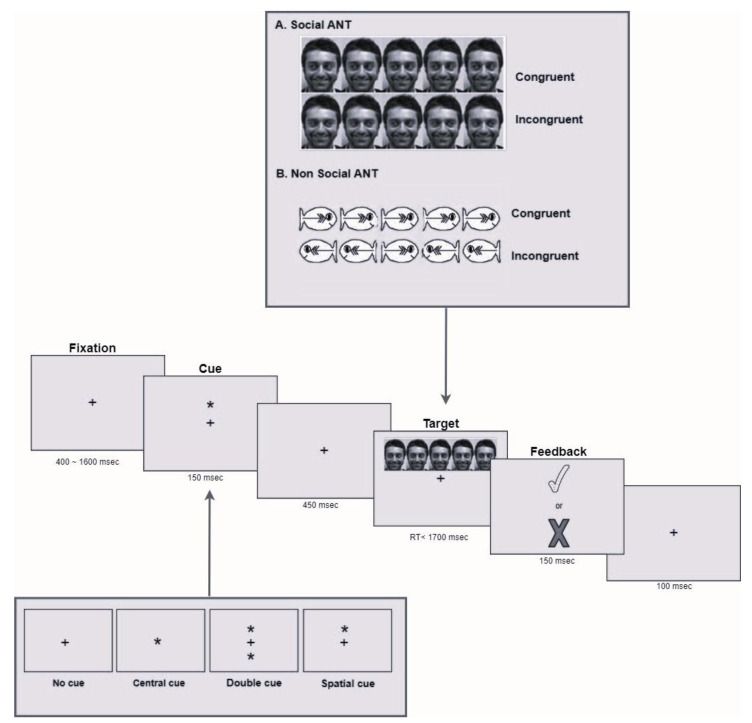
Detailed illustration of the modified version of the Attention Network Test (ANT). Modified version of Figure 1 in Federico and collaborators’ [22]. + = fixation cross; * = cue (central, double or spatial). Target stimulus and response feedback are reported.

The stimuli and trial sequences are illustrated in Figure 1. Each participant completed two different versions of the ANT that differed only in the types of stimuli presented. The non-social version presented colored fish as target and flanker stimuli [26], whereas the social version used photographs of human faces, as described by Federico and collaborators [25]. The target array consisted of a central stimulus and four flanker stimuli. Each stimulus subtended 1.6° (visual angle degree), and adjacent stimulus contours were separated by 0.21°. All five stimuli subtended a total of 8.84°. The target was presented about 1° above or below the fixation. Each target stimulus was preceded by one of four cue conditions: a center cue, a double cue, a spatial cue, or no cue. Each cue subtended 1.5° of the visual angle. Positive auditory and visual feedback on the performance was provided through an animation showing the target fish blowing bubbles or a red smile on the human face. Incorrect responses were followed by a single tone and no animation.

#### 2.2.3. Procedure

During the experimental session, the participants had to complete the two versions of the ANT (social and non-social). The administration order of the two tasks was randomized across participants. Each task consisted of a practice block with 24 trials and two experimental blocks of 48 trials each. The same instructions were provided for both tasks. Participants were told to look at the screen, on which social (photographs of human faces) or non-social (fish) stimuli would appear. Each target stimulus was preceded by a cue that served either to alert or orient the participants to the upcoming target. There were four cue types: no cue (neither an alerting nor an orienting cue was presented), a double cue (a double-asterisk cue appearing simultaneously above and below fixation; alerting), a spatial cue (a single asterisk presented in the upcoming target position; orienting), and a central cue (a single asterisk presented at the fixation cross location). After the cue, the target stimulus appeared and was flanked by one of two flanker types: congruent (flankers in the same direction as the target) or incongruent (flankers in the opposite direction to the target). To complete the task, the participants had to press the mouse button matching the direction in which the face was looking or the fish was facing. Participants were instructed to respond as quickly and accurately as possible. Each trial began with a fixation period of random variable duration between 400 and 1600 ms. A cue was presented for 150 ms. After cue disappearance, there was a short fixation period of 450 ms. Then, both the stimulus target and flanker appeared simultaneously. This visualization lasted until the participant’s response was detected, up to a maximum of 1700 ms. After the response, the participant received auditory and visual feedback from the computer.

#### 2.2.4. Data Analysis

We used simple subtractions of reaction times (RTs) and accuracy scores (Accs) between conditions to assess the participants’ attentional abilities. Visual cues were used to separately assess orienting and alerting. Alerting effect was calculated by subtracting the mean RTs and Accs of the double-cue conditions from the mean RTs and Accs of the no-cue conditions. These differences were considered as evidence of the speeding effect of alerting on responses. Orienting effect was calculated by subtracting the mean RTs and Accs of the spatial-cue conditions from the mean RTs and Accs of the central-cue conditions. The participants were alerted to the imminent appearance of the target by both central and spatial cues, but only the spatial cue provided spatial information that served to orient their attention to the correct location. Executive-control effect was calculated through the so-called “conflict effect” by subtracting the mean RTs and Accs of the congruent flanking conditions from the mean RTs and Accs of the incongruent flanking conditions. When flankers were congruent, discrimination of the target stimulus was facilitated, whereas incongruent flankers had a distracting effect on participants [4,5,22,25,27].

### 2.3. Statistical Analysis

Participants’ RTs and Accs were separately analyzed for each test output. A 2 × 3 mixed-model ANOVA was used to detect the effects of social processing and role type. Type of stimulus (social or non-social) was considered as a within factor, while role (defender, midfielder, or striker) was considered as a between factor. Each attention network was considered as a dependent variable. Post hoc analysis using the Bonferroni test was performed; 95% confidence interval (CI) lower (LCI) and upper (UCI) bounds were also considered. All analyses were conducted using JASP software version 16.04.

## 3. Results

### 3.1. Stimulus × Role Effects on Reaction Times (RTs)

**Figure 2:**
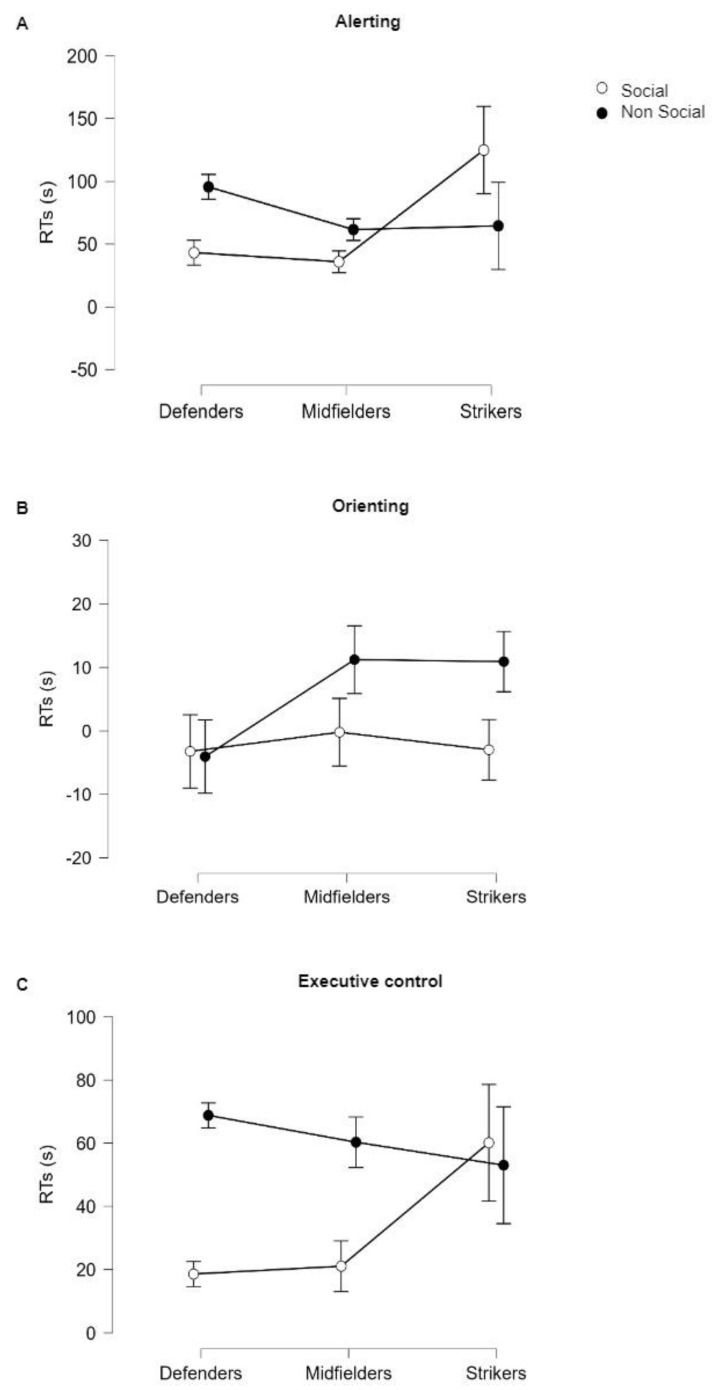
RT variations in (**A**) Alerting (double-cue vs. no-cue conditions); (**B**) Orienting (spatial-cue vs. central-cue conditions); and (**C**) Executive Control (congruent-flanking vs. incongruent-flanking conditions) are reported. On the y-axis RTs (s) and on the x-axis three-level between-factor Roles (Strikers, Midfielders, and Defenders) and two-level within-factor Stimuli (Social and Non-Social) are reported with separate lines. Abbreviations: RTs = reaction-time variations.

The results are shown in Table 2. Regarding the alerting network, no significant effect was found for Stimulus (F_1,99_ = 0.092; *p* = 0.762) or Role (F_2,99_ = 1.791; *p* = 0.172) or their interaction (F_2,99_ = 2.109; *p* = 0.08) (Figure 2A). Similarly, the orienting network showed no significant effect for Stimulus (F_1,99_ = 3.604; *p* = 0.061), Role (F_2,99_ = 0.888; *p* = 0.416), or their interaction (F_2,99_ = 1.109; *p* = 0.334) (Figure 2B). Finally, differences were found for executive-control networks. A significant effect was found for Stimulus (F_1,99_ = 6.761; *p* = 0.011 *). Post hoc Bonferroni testing revealed significant differences between social and non-social stimuli (t = −2.600; *p*_bonf_ = 0.011 *; mean difference = −27.464; LCI = −48.422; UCI = −6.506). No significant effect was found for Role (F_2,99_ = 1.081; *p* = 0.343). A significant effect was found for Stimulus × Role interaction; however, it did not survive post hoc Bonferroni testing (t = 2.636; *p*_bonf_ = 0.093; mean difference = 88.943; LCI = −11.303; UCI = 189.189) (Figure 2C).

### 3.2. Stimulus × Role Effects on Accuracy (Acc)

**Figure 3:**
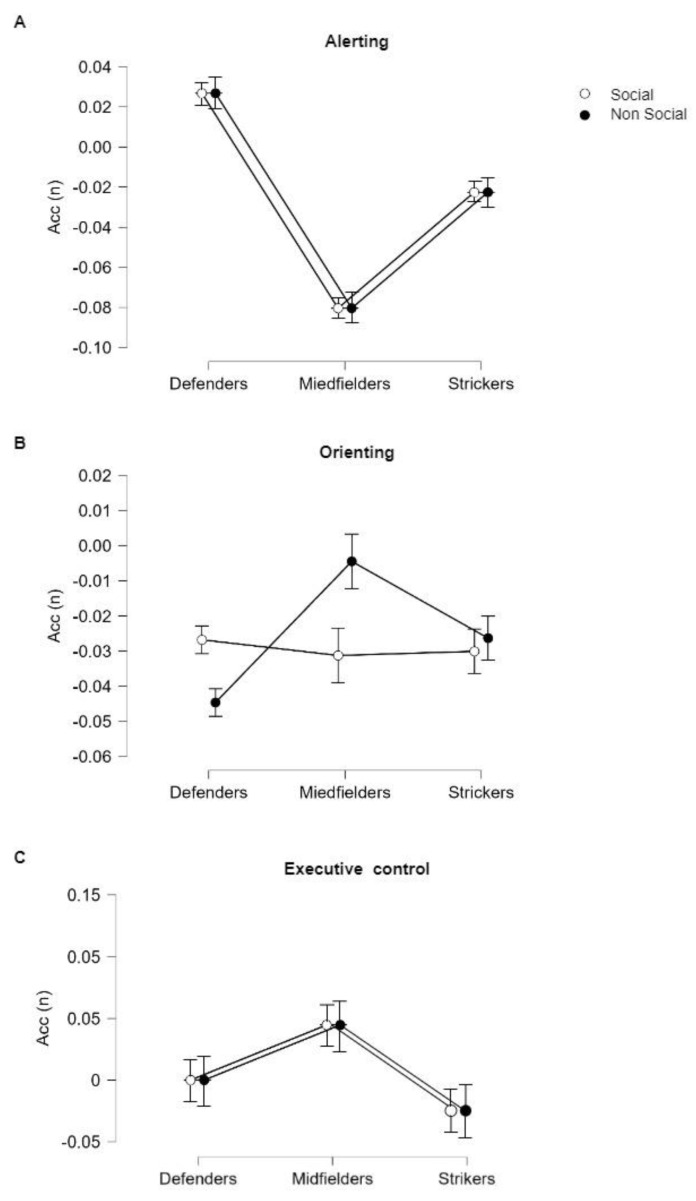
Accuracy variations in (**A**) Alerting (double-cue vs. no-cue conditions); (**B**) Orienting (spatial-cue vs. central-cue conditions); and (**C**) Executive Control (congruent-flanking vs. incongruent-flanking conditions) are reported. On the y-axis accuracy scores (n) and on the x-axis three-level between-factor Roles (Strikers, Midfielders, and Defenders) and two-level within-factor Stimuli (Social and Non-Social) are reported with separate lines. Abbreviations: Acc = accuracy score variations.

The results are shown in Table 3. As for the alerting network, differences were found between players. A significant effect was found for Role (F_2,99_ = 17.306; *p* < 0.001 ***). Post hoc Bonferroni testing revealed significant differences in Accs between Strikers and Midfielders (t = 3.303; *p*_bonf_ = 0.004 **; mean difference = 0.058; LCI = −48.422; UCI = −6.506); Striker and Defenders (t = −2.820; *p*_bonf_ = 0.017 *; mean difference = −.049; LCI = −0.092; UCI = −0.007); and Midfielders and Defenders (t = −5.876; *p*_bonf_ < 001 ***; mean difference = −0.107; LCI = −0.152; UCI = −0.063). No significant effect was found for Stimulus or Stimulus × Role interaction (Figure 3A). Regarding the orienting network, no significant effect was found for Stimulus (F_1,99_ = 0.684; *p* = 0.410) or Role (F_2,99_ = 0.750; *p* = 0.475). A significant effect was found for Stimulus × Role interaction (F_2,99_ = 6.022; *p* = 0.003 **). However, after post hoc Bonferroni test correction this effect was no longer present (t = −2.944; *p*_bonf_ = 0.061; mean difference = 0.013; LCI = −0.034; UCI = 0.041) (Figure 3B). Finally, the executive-control network showed no differences in accuracy between conditions (Stimulus: F_1,99_ = 0.074; *p* = 0.687) and players (Role: F_2,99_ = 0.429 *p* = 0.635). A significant effect was found for Stimulus × Role interaction (F_2,99_ = 7.089; *p* = 0.024 *). Again, after post hoc Bonferroni test correction this effect was no longer present (t = −3.456; *p*_bonf_ = 0.071; mean difference = 0.004; LCI = −0.005; UCI = 0.010) (Figure 3C).

## 4. Discussion

This study examined how social processing influences attentional abilities in team ball (or team sports) athletes playing different roles. Soccer, handball, and basketball athletes were selected since their playing conditions change constantly, forcing them to focus simultaneously on several environmental stimuli and thus modulate their attention levels to achieve better performance [6]. While scientific evidence indicates that attentional processes are crucial for these sports, how decisive elaborating stimuli with social value are remains to be investigated. Our study aimed to fill this gap by evaluating how social and non-social stimuli influence alerting, orienting, and executive-control attention networks. We chose to analyze three categories of roles: one mainly based on defense (defenders), one mainly based on tactics (midfielders), and one mainly based on attack (strikers). Differences between each of the player categories have been highlighted by Montuori and collaborators [10]. In their study, it was determined that the diversity of role performance relies on different cognitive processing modes and that a specific ‘executive profile’ could be developed based on the degree of cognitive flexibility needed for each of the three categories. Like cognitive flexibility, attention is a key component of executive functions, and the same is true for social attention, as different roles require different attentional abilities, which are fundamental components of executive functioning, just like cognitive flexibility.

To fully understand our results, it is necessary to remember the attention network approach, which considers the attention components as three functionally and anatomically distinct networks: alerting, orienting, and executive attention [2,28]. As previously mentioned, the alerting network is concerned with the ability to enhance sensitivity to incoming information and maintain it, the orienting network manages the ability to select and focus on the stimulus that needs to be attended to, and the executive-control network manages the ability to control behavior in order to achieve goals and resolve conflicts between alternative responses [25]. The Attention Network Test (ANT) [4] measures how exposure to stimuli that trigger or direct our attention speeds up and improves the accuracy of our behavioral responses. Further, using matching or contrasting flanker stimuli, with the ANT it is possible to measure how much the environment can influence our attentional focus and our capacity to ignore distractions. Owing to these characteristics, the ANT is suitable for studying alerting, orienting, and executive attention. In this context, Federico and collaborators [25] have proven that exposure to real faces positively affected the efficiency of executive control. Using a modified version of the ANT—with three different types of stimuli: fish, drawn faces, and photographs of faces—the authors demonstrated that behavioral evidence of cognitive interference (i.e., a slower reaction time to incongruent relative to congruent stimuli) was present only when fish and drawn faces were used, and not when photos of faces were used. These findings proved that exposure to social stimuli decreases cognitive interference and enhances the efficiency of executive control [29].

In our study, we used a similar protocol to prove that all athletes showed enhanced executive control when exposed to social stimuli, irrespective of the role played. The differences between RTs in congruent and RTs in incongruent flanking conditions were higher when the distracting stimuli were non-social (fish) rather than social (photographs of human faces) (Figure 2C). This finding once again demonstrates how social processing affects attention networks. According to Shults [30], faces are the most important source of social information (including the identity of a person, expression, gaze direction, age, and gender), and they are crucial for establishing social interaction. Several studies have demonstrated how faces are more likely to capture attention than other objects [31,32]. Moreover, it has been shown that after seeing a face with an averted gaze, one’s own attention will shift in the same direction of the seen gaze [33,34,35]. However, in everyday life, people often encounter conflicting gaze information from multiple faces. Thus, controlling how gaze information influences cognition is crucial for successful decision making, a cognitive process that is highly correlated with high levels of performance [25]. This evidence is in line with our findings, since our sample included team ball sports athletes for whom successful performance requires not only to be in touch with fellow team-mates but also to not be distracted by opponents. Interestingly, in our study, athletes showed no changes in alerting and orienting networks when faced with social stimuli. It might be implied that with the unexpected external environment surrounding the athletes during the game, they remain responsive to any kind of stimulus. As we discussed before, examining elements of the game environment is an essential skill for athletes. Among these elements, gaze direction seems to play a particular role. Riechelmann and collaborators [36] showed that individuals could anticipate others’ movements by analyzing their gaze direction. Martell and Vickers [37] found that others’ gaze direction influenced experienced athlete decision-making processes. Extensive amounts of research have now demonstrated that athletes are able to predict how events will unfold based on the movements of their opponents and that they use these predictions to increase the speed and accuracy of their decisions [38]. This ability has been associated with the functioning of the mirror-neuron system, a potential neural mechanism by means of which we understand other’s action goals [39]. As proven by multiple studies [40,41], athletes possess better-trained mirror-neuron systems and are able to easily predict other’s actions through the processing of certain key elements, among which gaze might be included.

Our second set of results showed that athletes’ performance accuracy was not influenced by the social or non-social nature of the stimulus. However, differences were found between athletes in different roles, specifically in alerting networks (Figure 3A). Both defenders and strikers improved their performance when the alert cue was shown before the target stimulus appeared. Conversely, midfielders’ performance remained the same with or without the appearance of the alert cue. Midfielders are the most tactical players in the game, as they are the ones who set the action in motion and have to deal with unexpected events and constantly change plans of attack and defense. Montuori and collaborators [10] showed that midfielders were the most flexible players, as they performed better in task-switching tests, while Schumacher and collaborators [42] found that midfielders outperformed defenders and strikers in simple reaction tasks. In addition to developing specialized physical skills, athletes who play different roles must also develop specific cognitive abilities [10]. According to this view, athletes of different roles employ attention networks in specific ways. Defenders and strikers—who are used to responding—react more to any type of information from their teammates or opponents, while midfielders—who are used to starting the action first—maintain their responsiveness and attentional focus in order to be always prepared to react and change their action plans.

Although our study presents novel insights, it has some limitations. Within our sample, we tested athletes from different sports, which, although similar, show a few differences in game tactics and team dynamics. Moreover, the striker group showed great variability in executive control in both social and non-social protocols. Future research should address these issues by investigating the specific attentional profiles of athletes with different roles within the same sports discipline. Our stimuli (social and non-social) also differed in terms of visual features; these differences should be addressed in future research. Further factors worth investigating could be gender differences and training experience.

## 5. Conclusions

According to our results, social stimuli play an important role in performance enhancement for athletes with open skills. We showed that social stimuli allow individuals to focus more on relevant information and ignore confounding background information. In addition, we demonstrated that it is possible to draw specific attentional profiles for different types of athletes. Our results showed that playing a strategic role (i.e., midfielder) enables an individual to acquire specific attentional skills that relate to the state of alertness. A study like ours could contribute to scientific research on social attention engagement in sports practice and its use as a training tool for athletes. Further research could focus on implementing these abilities in training protocols and outlining specific attention profiles for different types of sports. Finally, the influence of individual differences, such as gender, age, and training experience, should be investigated as well.

## Figures and Tables

**Table 1:** Participants’ demographics (number, age, BMI) are reported for each group.

	No. (n)	Age (Mean ± SD)	BMI (Mean ± SD)
Strikers	38	21.01 ± 3.61	23.73 ± 1.43
Midfielders	32	20.03 ± 3.23	23.55 ± 2.34
Defenders	32	18.50 ± 1.34	22.75 ± 1.45

**Table 2:** The 2 × 3 mixed ANOVA outputs for RTs. Bold values represent significant (* *p* < 0.05) results.

	Factors	F_(df)_	*p*
**Alerting**	Stimulus	0.092_(1,99)_	0.762
	Role	1.791_(2,99)_	0.172
	Stimulus × Role	2.109_(2,99)_	00.080
**Orienting**	Stimulus	3.604_(1,99)_	0.061
	Role	0.888_(2,99)_	0.416
	Stimulus × Role	1.109_(2,99)_	0.334
**Executive Control**	Stimulus	6.761_(1,99)_	0.011 *
	Role	1.081_(2,99)_	0.343
	Stimulus × Role	2.921_(2,99)_	0.059

**Table 3:** The 2 × 3 mixed ANOVA outputs for Acc. Bold values represent significant (* *p* < 0.05; ** *p* < 0.01; *** *p* < 0.001) results.

	Factors	F_(df)_	*p*
Alerting	Stimulus	0.087_(1,99)_	0.923
	Role	17.306_(2,99)_	<0.001 ***
	Stimulus * Role	0.098_(2,99)_	0.751
Orienting	Stimulus	0.684_(1,99)_	0.410
	Role	0.750_(2,99)_	0.475
	Stimulus * Role	6.022_(2,99)_	0.003 **
Executive Control	Stimulus	0.074_(1,99)_	0.687
	Role	0.429_(2,99)_	0.635
	Stimulus * Role	7.089_(2,99)_	0.024 *

## Data Availability

Data are available upon request from the corresponding author.
